# Impact of extreme wildfires on the geotechnical properties of volcanic soils: A specific dataset from Central-Southern Chile

**DOI:** 10.1016/j.dib.2025.111749

**Published:** 2025-06-02

**Authors:** Eduardo Javier Paredes-Delgado, Ignacio Pérez-Rey, Juan López-Vinielles, Roberto Tomás, Mauro Muñiz-Menéndez, Pablo Miranda, Roberto Sarro

**Affiliations:** a Faculty of Engineering, Civil Engineering Department, University of Santiago of Chile (USACH), Chile; b CINTECX, GESSMin Group, Department of Natural Resources and Environmental Engineering, Universidade de Vigo, s/n, Campus Universitario Lagoas-Marcosende, Vigo 36310, Spain; c Department of Geohazards and Climate Change, Geological and Mining Institute of Spain (IGME-CSIC), Ríos Rosas 23, Madrid 28003, Spain; d Department of Civil Engineering, University of Alicante, Alicante P.O. Box 99, 03080, Spain; e Laboratorio de Geotecnia - CEDEX, Madrid 28014, Spain

**Keywords:** Chile, Extreme wildfires, Geotechnics, Volcanic soils

## Abstract

The increasing occurrence of wildfires, driven by climate change and environmental degradation, highlights the importance of studying their effects from the perspective of geotechnics and soil mechanics. During a wildfire in 2023 in Central-Southern Chile, temperatures in eucalyptus forests reached up to 600 °C, resulting in significant physical and chemical alterations in the soil. This dataset focuses on the impact of wildfires on the geomechanical properties of volcanic soils in central Chile, providing data collected through laboratory tests and *in-situ* survey techniques. The methods employed include: (i) delineation of areas severely affected by the 2023 wildfires in the Biobío region of Chile; (ii) measurement of soil geomechanical parameters in affected areas and comparison with pre-fire values; and (iii) compilation of a dataset to facilitate the evaluation of post-fire changes in soil geomechanical properties. This dataset can serve as a reference for assessing the impacts of wildfire-induced changes in soils, providing valuable insights for identifying areas prone to ground instability and informing civil engineering projects.

Specifications Table

**Table:** 

Subject	Geotechnical Engineering and Engineering Geology
Specific subject area	Geotechnical properties of volcanic soils
Type of data	Table, Image, Graph and Figure. Raw and analyzed.
Data collection	The volcanic soil samples analyzed in this study were collected during comprehensive field investigations. Sampling sites were precisely determined by delineating the perimeters of the 2023 extreme wildfire in Chile using high-resolution Sentinel-2 satellite imagery. Soil pits were excavated at eight locations, with depths reaching up to 1.5 m. *In-situ* tests conducted included Dynamic Cone Penetrometer (DCP), density measurements, and hydraulic conductivity assessments. Laboratory tests on soil samples included particle size distribution, modified Proctor compaction, California Bearing Ratio (CBR), unconfined compression tests, pH measurement, and loss on ignition. These tests were conducted following precise sample preparation and protocols aligned with international standards, using exclusively standardized equipment.
Data source location	Región del Biobío, Provincia de Biobío, Chile.
Data accessibility	Repository name: Impact of extreme wildfires on the geotechnical properties of volcanic soils: A specific dataset from Central-Southern Chile Data identification number: 10.5281/zenodo.15198554 Direct URL to data: https://zenodo.org/records/15392078
Related research article	None

## 1 Value of the Data

• This unique dataset provides a reference for understanding the effects of wildfires on the geomechanical properties of volcanic soils, providing valuable baseline data for researchers studying soil behavior under high temperature in natural conditions.
• The dataset includes detailed measurements of the physical, chemical, and mechanical properties of volcanic soils from both burned and unburned areas, allowing for comparative analyses and investigation on the impacts of wildfires on soil properties.
• This dataset can be useful for evaluating load-bearing capacities and assessing slope instability in areas affected by wildfires, supporting engineering solutions and hazard assessments in post-fire landscapes.
• By providing a comprehensive dataset of wildfire-induced changes in volcanic soils, this resource facilitates further research into the adverse geotechnical effects of wildfires and their potential contribution to ground movements.
• The geotechnical information provided in this dataset supports civil engineering project planning and contributes to the development of risk mitigation strategies based on geotechnical principles in regions affected by wildfires.

## 2 Background

Volcanic soils in central and south-central Chile, derived from pyroclastic materials such as ash, sand, and pumice, show diverse topographies and cover extensive areas [1]. Although they share a common volcanic origin, they encompass four distinct soil types, locally known as Trumaos, red-clay soils, brown-clay soils and Ñadis [2], are found between the Biobío region and parts of Aysén. They originate from the degradation of pyroclastic materials mixed with organic matter from native forest debris. Due to their mineralogical composition, dominated by allophane and halloysite, these soils are characterized by a high-water retention capacity and significant sensitivity to environmental changes. Given their high variability, assessing the geotechnical properties of Trumaos, and particularly replicating their natural conditions in laboratory tests is challenging, which makes the establishment of standardized parameters highly complex.

Wildfires during 2022–2023 affected over 417,809 ha in Chile, with 43% of the burned area located in the Biobío region [3]. These events highlighted the need to study the impacts of wildfires on volcanic soils [4,5]. This dataset was compiled to provide detailed information on the geomechanical properties of volcanic soils before and after wildfire exposure, thereby facilitating further research and practical applications.

## 3 Data Description

The dataset (10.5281/zenodo.15392078) includes a wide range of geotechnical properties to understand the effects of extreme wildfires on volcanic soils, providing relevant information for the planning and execution of recovery projects in wildfires-affected areas.

The dataset is divided into *in-situ* and laboratory tests, which collectively assess the physical, chemical, and mechanical properties of the studied soils. *In-situ* tests include density, penetration, and permeability tests. Laboratory tests comprise granulometric analysis, liquid and plastic limit tests, particle density determination, modified Proctor tests, California Bearing Ratio (CBR), unconfined compression tests, loss on ignition (LOI) tests, and pH determination. All these properties provide critical information on the structural, mechanical, and compositional characteristics of volcanic soils, highlighting how they change after a wildfire. These changes should be taken into account when planning civil engineering projects in burned areas [6], as well as for assessing geohazards, particularly those related to slope instabilities [7].

The data obtained from each analysis are presented and described in dataset “Impact of extreme wildfires on the geotechnical properties of volcanic soils: A specific dataset from Central-Southern Chile”. The directory structure of the dataset is as follows:

• Test Records. This folder compiles all the documentation related to the geotechnical testing activities carried out. The tests are categorized into two main groups: laboratory tests and *in-situ* tests.
• Test Graphs. This folder includes graphical representations of the results obtained from both laboratory and *in-situ* tests. These graphs make it easier to visualize parameters, trends, and correlations that complement the numerical data recorded in the test forms.
• Other information. This folder contains the stratigraphic profiles derived from borehole and test pit data, as well as some photographs that help illustrate the work that has been performed.

Table 1 provides a summary of the techniques and methodologies used for the geotechnical characterization of the studied soils, along with their corresponding standards. These methods encompass both *in-situ* and laboratory tests, enabling a comprehensive evaluation of the physical, chemical, and mechanical properties of the soils before and after wildfire exposure.

**Table 1:** Techniques/methods and associated standard.

Method /Technique	Associated Standard Highway Manual	ISO or Equivalent Reference
*In-Situ* Density	M.C. V8 8.102.9 – Sand Cone Method for Determining *In-Situ* Density	ISO 11272:2017 ASTM D1556/D1556M-15 UNE 103501-1:2000
Soil Sampling	M.C. V8.3.100 – Soil and Rock Sampling for Road Engineering Projects	ISO 18400-101:2017 UNE-ISO 10381-1:2007
Penetration Tests (DCP)	M.C. V8.102.12 – Portable Dynamic Cone Penetrometer (DCP) Test Method	ISO 22476-1:2022 ASTM D6951/D6951M-18 UNE-EN ISO 22476-2:2008/A1:2014
Permeability Tests	Geotechnics - Determination of saturated hydraulic conductivity in the field – Variable head test or Porchet test (NCh3610:2020)	ISO 22282-2:2012 UNE 103501-3:2000
Grain-Size Distribution Tests	M.C. V8 8.102.1 – Particle Size Distribution Test Method	ISO 11277:2020 ASTM D6913/D6913M-1 UNE EN 933-1:2012
Liquid Limit Test	M.C. V8 8.102.3 – Method for Determining the Liquid Limit (Casagrande Method)	ISO 17892-12:2018/Amd 2:2022 ASTM D4318-17 UNE-EN ISO 17892-12:2019
Plastic Limit Test	M.C. V8 8.102.4 – Method for Determining the Plastic Limit	
Modified Proctor Test	M.C. V8 8.102.7 – Method for Determining the Moisture-Density Relationship – Modified Proctor Test	ASTM D1557-12(2021) UNE 103501:1994
Determination of Particle Density (Specific Gravity) of Soil Solids Test	M.C. V8 8.102.10 – Method for Determining the Specific Gravity of Soil Solids	ISO 11508:2017 ASTM D854-23 UNE 103301:1994
California Bearing Ratio (CBR)	M.C. V8 8.102.11 Method for Determining the California Bearing Ratio (CBR)	ASTM D1883-21 UNE 103502:1994
Unconfined Compressive strength Test	Soil mechanics - Test methods - Determination of unconfined compressive strength of cohesive soils (NCh3134:2007)	ISO 17892-7:2017 ASTM D2166/D2166M-16 UNE 103400:1994
Loss on Ignition Test	Recommended soil analysis methods for Chile - 7.2 Loss on ignition [9]	ISO 10694:1995 ASTM D2974-00 UNE-EN 17685-1:2023
pH Determination Test	Soil Quality – pH Determination (NCh3414:2016)	ISO 10390:2005 ASTM D4972-19 UNE-EN ISO 10390:2022

Most of the techniques and methodologies applied in this study are based on the guidelines established in the Highway Manual of the Chilean Ministry of Public Works. These guidelines are publicly accessible online [8], provide detailed descriptions of the methods, and are widely adopted for various soil and rock testing applications in Chile. Moreover, the majority of these procedures have equivalent or closely related standards under ISO or other internationally recognized frameworks (see Table 1).

Additionally, three of the methods employed in this study are regulated by national standards issued by the Instituto Nacional de Normalización (INN), the Chilean National Institute for Standardization. In these cases, a brief description of the corresponding methodologies is provided below:

• Geotechnics – Determination of Saturated Hydraulic Conductivity in the Field – Variable Head Test or Porchet Test (NCh3610:2020): This method involves excavating a cylindrical pit in which a constant water head is maintained and monitored. The infiltration rate is measured over time, allowing the calculation of the saturated hydraulic conductivity. This technique is commonly used to assess the permeability of natural soils under field conditions.
• Soil Mechanics – Test Methods – Determination of Unconfined Compressive Strength of Cohesive Soils (NCh3134:2007): This test involves preparing cylindrical samples of cohesive soil, which are subjected to axial compression without lateral confinement until failure occurs. The maximum axial stress recorded during the test corresponds to the unconfined compressive strength of the material.
• Soil Quality – pH Determination (NCh3414:2016): This method follows a potentiometric approach, where a soil sample is mixed with distilled or deionized water in a specified ratio. After equilibrium is reached, the pH of the suspension is measured using a calibrated pH meter. This test is used to evaluate the chemical reactivity and potential biological activity of the soil.

The standardization of these procedures ensures the reproducibility and reliability of the results, facilitating comparative analyses and supporting engineering applications in fire-affected areas.

## 4 Experimental Design, Materials and Methods

### 4.1 Soil sampling

**Fig. 1:**
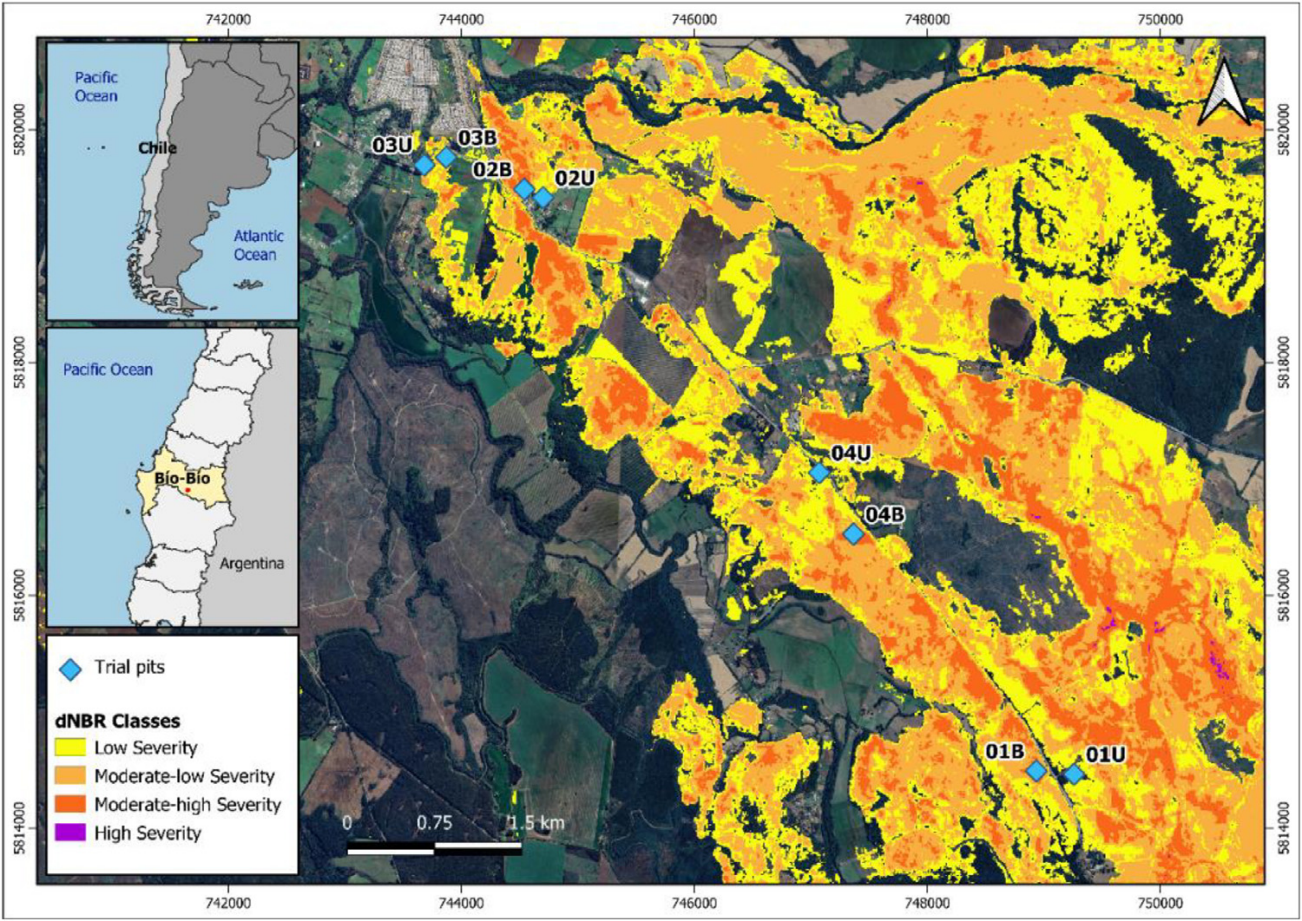
Location of the study area and wildfire map obtained by dNBR Index. The blue diamonds indicate the location of the trial pits.

To collect the samples, areas affected by the extreme wildfire in Chile in 2023 were first identified using Sentinel-2 satellite imagery. The difference between the pre-fire and post-fire Normalized Burn Ratio (NBR) obtained from the satellite images was used to calculate the differential NBR (dNBR or ∆NBR; Key & Benson [10]), which distinguishes between vegetated areas (before the wildfire) and burned areas based on their spectral responses. Additionally, dNBR was reclassified following the classification scheme proposed by Keeley J.E. (2009) to map areas by burn severity. Using the delineated perimeter of the burned area (Fig. 1) and geological maps, sampling points were planned to include both fire-affected and non-fire-affected soils. Subsequently, the selected sampling points were validated in the field to ensure they met the requirements for conducting the tests.

At the selected sampling points, a geotechnical soil sampling survey was conducted, involving eight test pits (Fig. 1) excavated to a depth of 1.50 m. *In-situ* tests were also conducted at the sampling locations to determine characteristic soil parameters and complement the data obtained through laboratory testing. These *in-situ* tests, which included visual descriptions, stratigraphic analyses, hydraulic conductivity tests, *in-situ* density measurements, and penetration tests, provided essential data for subsequent laboratory analyses.

The fieldwork and *in-situ* tests performed are detailed in Table 2. All collected soil samples were obtained in accordance with the requirements outlined in Chilean Standard NCh3400/2:2016 [11]. This standard provides detailed guidelines on appropriate sampling methodologies to ensure the representativeness and integrity of soil samples. Following these protocols, the samples were used for laboratory testing (as presented in Table 3).

**Table 2:** Location and characteristics of each sampling site.

Trial pit	WGS 1984 Coordinate System, Zone 18S (UTM)		Depth [m]	Type	Severity according to Keeley [12]
	North	East			
01B	5814504	748940	1.50	Affected by wildfire	Moderate-high Severity
01U	5814464	749259	1.50	Natural condition	Unburned
02B	5819488	744541	1.50	Affected by wildfire	Moderate-high Severity
02U	5819416	744702	1.50	Natural condition	Unburned
03B	5819762	743871	1.50	Affected by wildfire	Moderate-low Severity
03U	5819625	743700	1.50	Natural condition	Unburned
04B	5816543	747374	1.50	Affected by wildfire	Moderate-high Severity
04U	5817069	747049	1.50	Natural condition	Unburned

**Table 3:** Field techniques and their objectives.

Field technique		Objective
Soil sampling	Unburned samples	Study soil conditions in their natural state
	Burned samples	Compare measurable soil parameters and characteristics with those of unburned samples to identify potential changes caused by fire.
*In-situ* soil testing	*In-situ* density	Determine bulk density and natural moisture content of the soil at different depths.
	Penetration test	Assess penetration resistance at different depths.
	Permeability test	Determine soil hydraulic conductivity at different depths

### 4.2 *In-situ* testing

The techniques employed for conducting the *in-situ* testing are presented in Table 3.

**Fig. 2:**
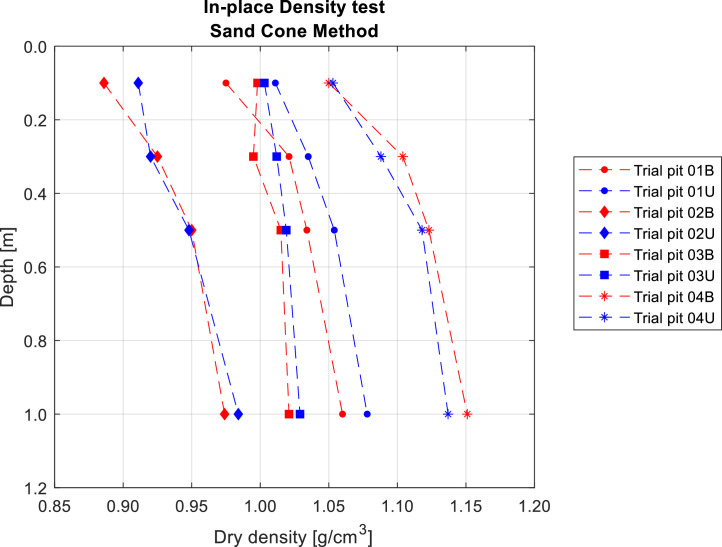
Determination of *in-situ* density at the eight sampling points. Note that the trial pits located in burned areas are plotted in red, while those placed in unburned areas are plotted in blue.

*In-situ* density tests using the sand cone method were performed at the following depths: 0.1, 0.3, 0.5 m and 1 m. Given that the soils under study were fine-grained, these tests were conducted without significant difficulties. The boreholes showed regular cross-sections, and material recovery from the excavation was relatively straightforward. The results are plotted in Fig. 2.

**Fig. 3:**
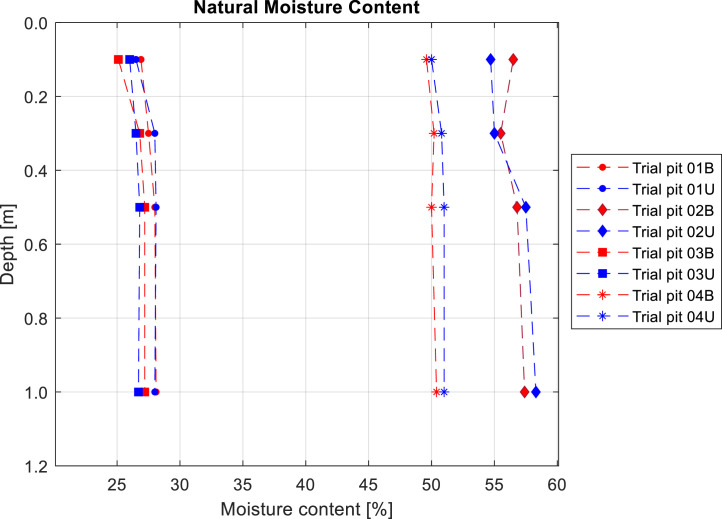
Determination of the natural moisture content of the soil at the eight sampling points. Note that the trial pits located in burned areas are plotted in red, while those placed in unburned areas are plotted in blue.

The determination of the natural moisture content complements the *in-situ* density measurement, enabling the calculation of the dry density of the soil. Soil samples for moisture content analysis were collected at the same depths as the *in-situ* density measurements, ensuring direct comparability. Additionally, sampling was conducted under consistent conditions to maintain uniformity. These depths were set as 0.1, 0.3, 0.5, and 1 m (Fig. 3).

**Fig. 4:**
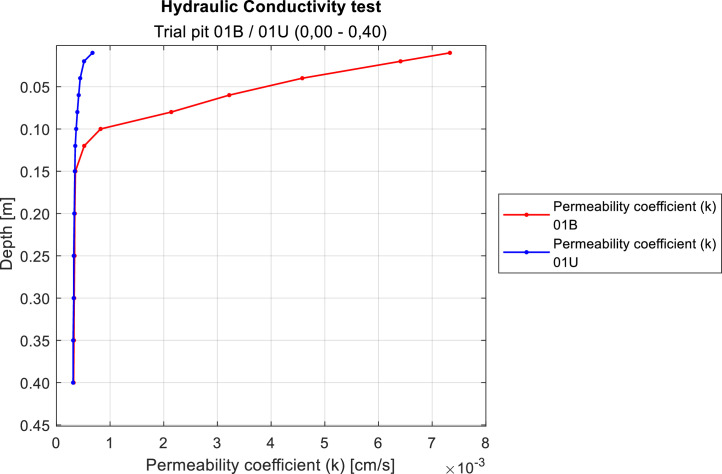
Determination of the natural moisture content of the soil in the trial pit 01U and 01B.

Given the low permeability of the studied soils, the method for determining hydraulic conductivity was adjusted in terms of test duration. The results presented in this study were obtained following the Chilean standard NCh3610:2020. This standard outlines the methodology for determining the field saturated hydraulic conductivity of soils. The standard 60 min timeframe proved insufficient to measure water absorption to a depth of 40 cm. During the excavation of the test pits, a horizontal platform was prepared with dimensions at least three times the diameter of the borehole required for the test. This step was taken to prevent distorted results due to insufficient soil volume. Measurements were recorded at fixed descent depths between 1 and 40 cm, with the recorded data representing the time taken to reach each depth (Fig. 4).

### 4.3 Laboratory tests

Laboratory tests conducted on soils samples aimed to determine the physical, mechanical, and chemical properties of the soil under controlled conditions. These tests enable a comparative analysis of soil properties between wildfires-affected and unaffected areas. Samples preparation posed a challenge due to the technical impossibility of oven-drying, intrinsic to the Trumaos, which are highly susceptible to significant changes in their properties when subjected to any variation in their natural state caused by external factors. As a result, the samples were processed while maintaining their natural or controlled moisture levels and avoiding exposure to environmental conditions, thereby preserving the natural properties of the analyzed soils. Table 4 lists the laboratory tests performed.

**Table 4:** Laboratory work and objectives.

Technique		Application objectives
Soil physical characterization	Grain-Size Distribution	Determine soil particle-size distribution for classification purposes
	Atterberg limits	Determine consistency of fine-grained soils through the liquid limit, the plastic limit and the plasticity index and for classification purposes
	Determination of Particle Density (Specific Gravity) of Soil Solids	Determine soil specific gravity
	Modified Proctor test	Determine soil maximum dry compacted density and optimum moisture content
Soil mechanical characterization	Unconfined compressive strength test	Determine soil undrained shear strength
	California Bearing Ratio (CBR)	Determination of bearing capacity
Soil chemical characterization	Loss on ignition (LOI) test	Estimate organic matter content and soil behavior under calcination
	pH test	Determine pH at different depths and soil conditions.

**Fig. 5:**
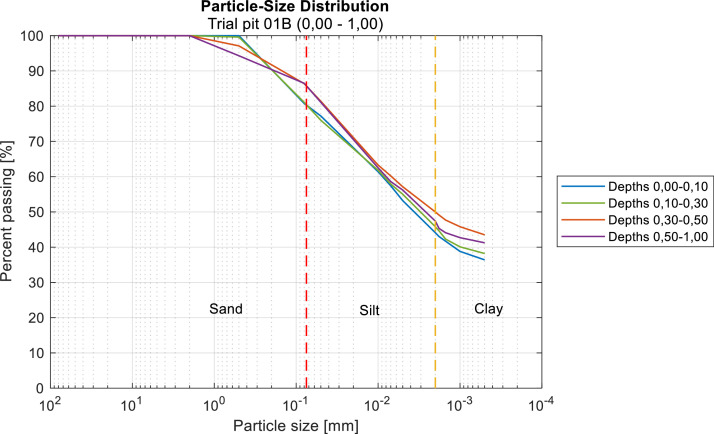
Granulometric analyses (curves) for trial pit 01B, placed in a burned area. The different types of soils have been classified according to ASTM (reference).

First, granulometric tests were conducted in the laboratory. Since the studied soils were predominantly fine-grained, the traditional granulometric sieve analysis method was used, complemented by sedimentation analysis for particles smaller than 0.08 mm. As an example of the results, the data from Test Pit 01B, located in a burned area, are shown in Fig. 5. Information from the other test pits is available in the repository specified in the **Specifications Table**.

**Fig. 6:**
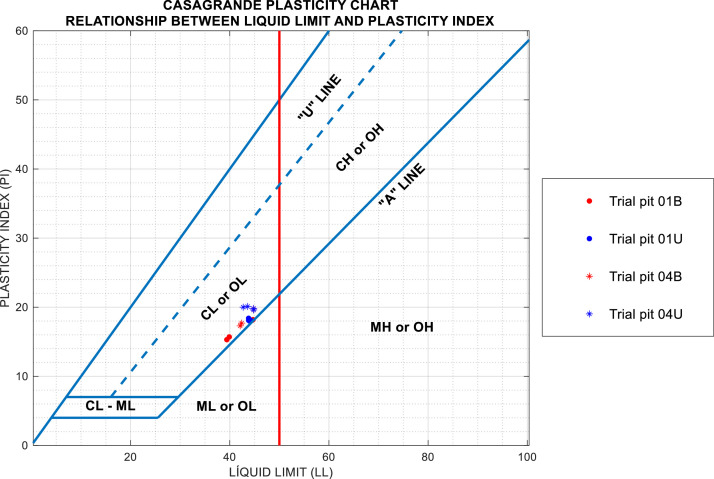
Representation of soils from trial pits 01B, 01U, 04B, and 04U on Casagrande's plasticity chart. Note that soils samples located in burned areas are plotted in red, while those placed in unburned areas are plotted in blue.

Additionally, Atterberg limits tests were conducted to assess the plasticity characteristics of soil samples from both wildfire-affected and unaffected areas, including the liquid limit (LL), plastic limit (PL), and plasticity index (PI). Fig. 6 presents the results for trial pits 01B, 01U, 04B, and 04U, showing the variations in these parameters.

**Fig. 7:**
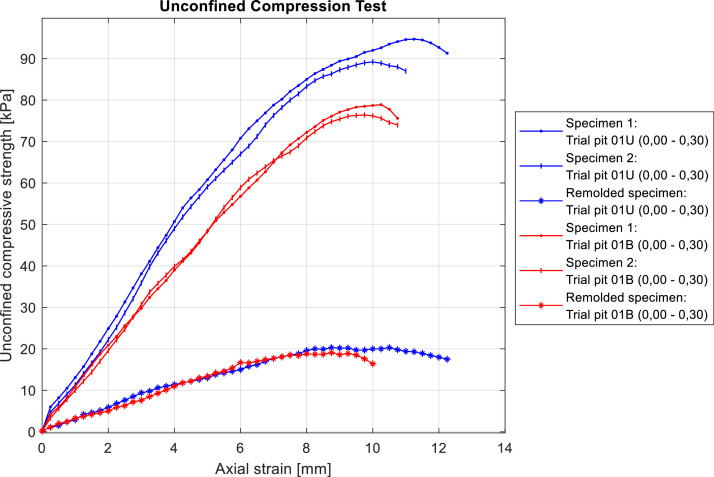
UCS test stress-strain curves for the six specimens tested. Soils samples located in burned are plotted in red, while those placed in unburned areas are plotted in blue.

The undrained shear strength of the studied soils was determined from unconfined compression tests results. Six specimens, each 10 cm in height, were tested: four were extracted from the first 30 cm of trial pits 01U and 01B, while the remaining two were remolded from disturbed samples taken from the same trial pits. Based on these results, the sensitivity index of the soil was calculated (Fig. 7). It should be noted that the load was applied to produce an axial strain rate of 2.0%/min, in accordance with Chilean Standard NCh3134:2007 [13].

**Fig. 8:**
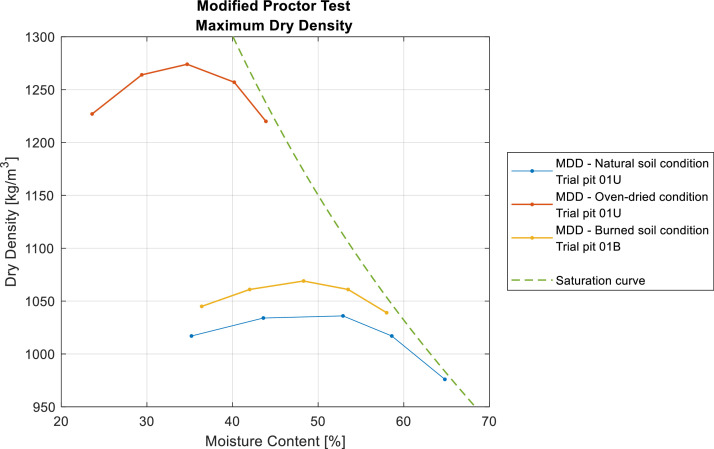
Maximum dry compacted density obtained under different soil conditions from trial pit 01U and 01B.

The modified Proctor compaction test was used to study the compactability of soils by analyzing the relationship between the moisture content of a soil and the dry density achieved when compacted at a given energy. The maximum dry compacted density was determined for three samples from two test pits at depths between 0.0 and 0.3 m. The samples were selected based on their natural moisture content, allowing the Proctor test to be conducted without prior oven drying. Three samples were tested: an unaffected sample with added moisture, an oven-dried sample, and a wildfire-affected sample with added moisture (Fig. 8).

**Fig. 9:**
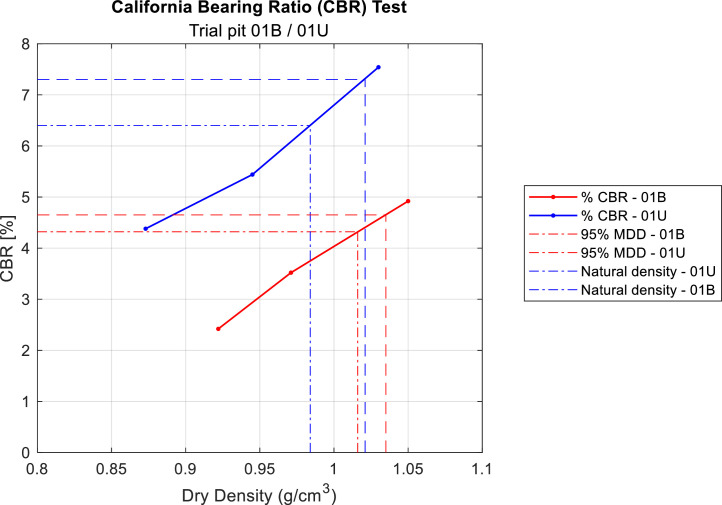
Maximum dry compacted density obtained in laboratory under different soil conditions from trial pit 01U and 01B for 0.00 to 0.30 m. Note that soils samples located in burned areas are plotted in red, while those placed in unburned areas are plotted in blue.

California Bearing Ratio (CBR) tests were conducted on samples collected from depths ranging from 0.0 to 0.3 m (Fig. 9) and from 0.3 to 0.5 m, including soils affected by the 2023 wildfire, and comparing them to unaffected soils.

**Fig. 10:**
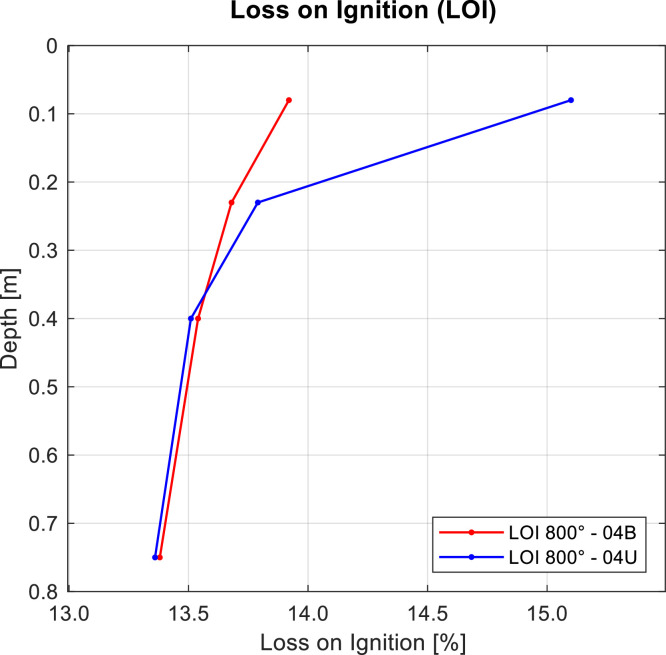
Results for LOI test at 800 °C for wildfire-affected and unaffected soil samples in trial pit 04U and 04B. Note that soil samples located in burned areas are plotted in red, while those in unburned areas are plotted in blue.

Loss on ignition tests were then conducted using the method described by [9], which involves drying the soil at a temperature of 105 ± 5 °C until a constant mass was achieved, followed by heating at 360 °C for 16 h. This method is commonly used to estimate the organic matter content of soil samples (ignition up to 800 °C). Fig. 10 shows the loss on ignition in the upper centimeters of the analyzed samples, comparing the results of wildfire-affected and unaffected soil samples.

**Fig. 11:**
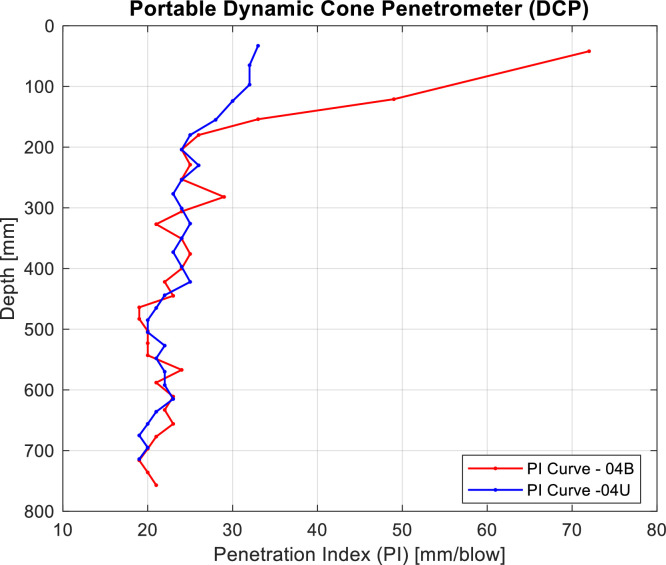
Evolution of Penetration index with depth for trial pit 04U and 04B. Note that soils samples located in burned areas are plotted in red, while those placed in unburned areas are plotted in blue.

DCP penetration tests were conducted near the excavated test pits, with two measurements per site to ensure the representativeness of the tested soil. The surface layer was left undisturbed, and areas with roots were avoided. Given the fine nature of the soil and its low resistance to penetration, measurements were recorded after each blow, with no irregularities observed. These tests produced characteristic penetration curves of the soil (Fig. 11), from which the Penetration Index (DN) was derived, illustrating the relationship between penetration index and depth. The device used in this study was a standard model featuring a 60° hardened steel cone (Ø 20 mm) and an 8 kg hammer dropped from a height of 575 mm, in accordance with the specifications of MC-V8 8.102.12 [8].

## Limitations

While this dataset provides valuable information on the geotechnical properties of volcanic soils before and after the extreme wildfires in Central-Southern Chile in 2023, its practical application in engineering decisions must carefully account for site-specific variables, project requirements, and other factors such as human activities. Furthermore, the datasets are focused on *Trumaos* soils and do not include other geological settings in the region. In this context, similar analyses are crucial for other soil types affected by this wildfire event, as well as for other lithologies in areas of Chile also affected by wildfires.

## Ethics Statement

The authors have read and followed the ethical requirements for publication in Data in Brief and confirm that the current work does not involve human subjects, animal experiments, or any data collected from social media platforms.

## CRediT authorship contribution statement

**Eduardo Javier Paredes-Delgado:** Conceptualization, Data curation, Formal analysis, Investigation, Methodology, Validation, Visualization, Writing – original draft. **Ignacio Pérez-Rey:** Formal analysis, Validation, Visualization, Writing – review & editing. **Juan López-Vinielles:** Methodology, Visualization, Writing – review & editing. **Roberto Tomás:** Validation, Writing – review & editing. **Mauro Muñiz-Menéndez:** Validation, Writing – review & editing. **Pablo Miranda:** Methodology, Visualization, Writing – review & editing. **Roberto Sarro:** Supervision, Conceptualization, Formal analysis, Investigation, Methodology, Validation, Writing – original draft.

## Data Availability

(Zenodo).Impact of extreme wildfires on the geotechnical properties of volcanic soils: A specific dataset from Central-Southern Chile (Original data)
